# Organic vs. Conventional Chestnuts (*Castanea sativa* Mill.): A Focus on Antioxidant Activity, Volatile Compounds, and Sensory Profile

**DOI:** 10.3390/foods14122013

**Published:** 2025-06-06

**Authors:** Maria Teresa Frangipane, Lara Costantini, Stefania Garzoli, Nicolò Merendino, Riccardo Massantini, Piermaria Corona

**Affiliations:** 1 Department for Innovation in Biological, Agro-Food and Forest Systems (DIBAF), University of Tuscia, via San Camillo de Lellis, 01100 Viterbo, Italy; massanti@unitus.it (R.M.); piermaria.corona@unitus.it (P.C.); 2 Department of Ecological and Biological Sciences (DEB), Tuscia University, Largo dell’Università snc, 01100 Viterbo, Italy; lara.cost@unitus.it (L.C.); merendin@unitus.it (N.M.); 3 Department of Chemistry and Technology of Drugs, Sapienza University of Rome, Piazzale Aldo Moro 5, 00185 Rome, Italy; stefania.garzoli@uniroma1.it; 4 Study Alpine Centre, University of Tuscia, Via Rovigo, 7, 38050 Pieve Tesino, Italy; 5 CREA Research Centre for Forestry and Wood, 52100 Arezzo, Italy

**Keywords:** quality, sensorial traits, antioxidants, chestnuts (*Castanea sativa* Mill.), volatile chemical composition, organic farming, CATA (Check-All-That-Apply) test

## Abstract

The consumption of organic foods is on the rise, as health-conscious consumers increasingly perceive them as superior both in nutritional value and for overall well-being. However, data on the nutritional and sensory properties of organic chestnuts remain scarce. This research aimed to evaluate and compare the nutritional and sensory characteristics of organic and conventional chestnuts. Results indicate that organic chestnuts exhibit a distinct sensory profile and achieve significantly higher overall scores in sensory analysis compared to conventional chestnuts. Specifically, organic chestnuts displayed stronger aromas of chestnut (9 vs. 8), hazelnut (5.87 vs. 5), almond (5 vs. 4), butter (3.96 vs. 3), and floral notes (5.95 vs. 4.96). Notably, organic chestnuts were strongly characterized by a caramel aroma, which was completely absent in conventional chestnuts (1.95 vs. 0), and probably due to the exclusive presence of decanal and 1-pentanol observed among the volatile compounds. Furthermore, organic chestnuts demonstrated a higher nutritional value, particularly in terms of antioxidant content. The total phenolic content (TPC) was significantly greater in organic chestnuts (6.54 mg GAE/g) compared to conventional samples (5.18 mg GAE/g). The relationships between attributes and consumers’ perceived liking revealed a strong association between liking and the attributes of caramel, floral, hazelnut, almond, and chestnut. These attributes are specific to organic chestnuts. As a result, both consumers and the trained panel prefer organic chestnut samples over conventional ones. Promoting the consumption of organic chestnuts by enhancing knowledge and awareness of their characteristics can encourage their use, contributing to key health and environmental sustainability goals.

## 1. Introduction

The area under organic farming in the European Union reached 14.8 million hectares in 2022, accounting for 20% of the global total. Organic production has experienced significant growth over the past decade [1] and is expected to continue expanding in the coming years. Among European countries in 2022, Italy led in the number of organic producers, with approximately 82,600, followed by Greece (58,600) and France (58,400) [1].

In Italy, the area dedicated to organic chestnut cultivation reached 18,300 hectares in 2022, marking a 7.2% increase compared to 2021 [2]. Organic farming is a crucial factor in the sustainability of agriculture and food systems. For some crops, such as cereals and soybeans, comparative analyses of the environmental impacts of conventional and organic farming have been conducted [3]. However, studies on tree crops remain relatively scarce [4]. At the same time, consumers are becoming increasingly aware of the importance of organic foods, which are perceived as safer, healthier, and more environmentally friendly than conventionally produced alternatives. Specifically, some studies have indicated that consumers are more likely to prefer organically grown chestnuts over conventionally grown ones [5]. Nonetheless, the significantly lower yields associated with organic production remain a major disincentive. This challenge must be counterbalanced by higher selling prices for organic products [6].

The nutritional and health benefits of including chestnuts in the human diet have been well-documented in the literature [7,8,9]. Due to their antioxidant and nutraceutical properties, there is growing interest in high-quality chestnut cultivars for their bioactive components [10,11,12]. Additionally, studies have examined the sensory profiles of different chestnut cultivars [13]. Our study aims to evaluate and compare the antioxidant activity, sensory profiles, and volatile chemical composition of organically grown chestnuts from Monti Cimini (Lazio, Italy) with those of conventionally grown chestnuts. It represents the first initiative to enhance the value and characterization of organic chestnuts based on their sensory and biomolecular attributes while contributing to the broader objective of environmental sustainability.

Within the framework of a circular economy, the study compares, in terms of nutritional and antioxidant properties, both conventional chestnuts (CCs), conventional chestnuts with pellicle (CCPs), organic chestnuts (OCs), and organic chestnuts with pellicle (OCPs). In fact, the pellicle, which is considered a byproduct, could provide additional value and further promote the sustainability and economic viability of organic chestnut production.

## 2. Materials and Methods

### 2.1. Sample Preparation

**Figure 1:**
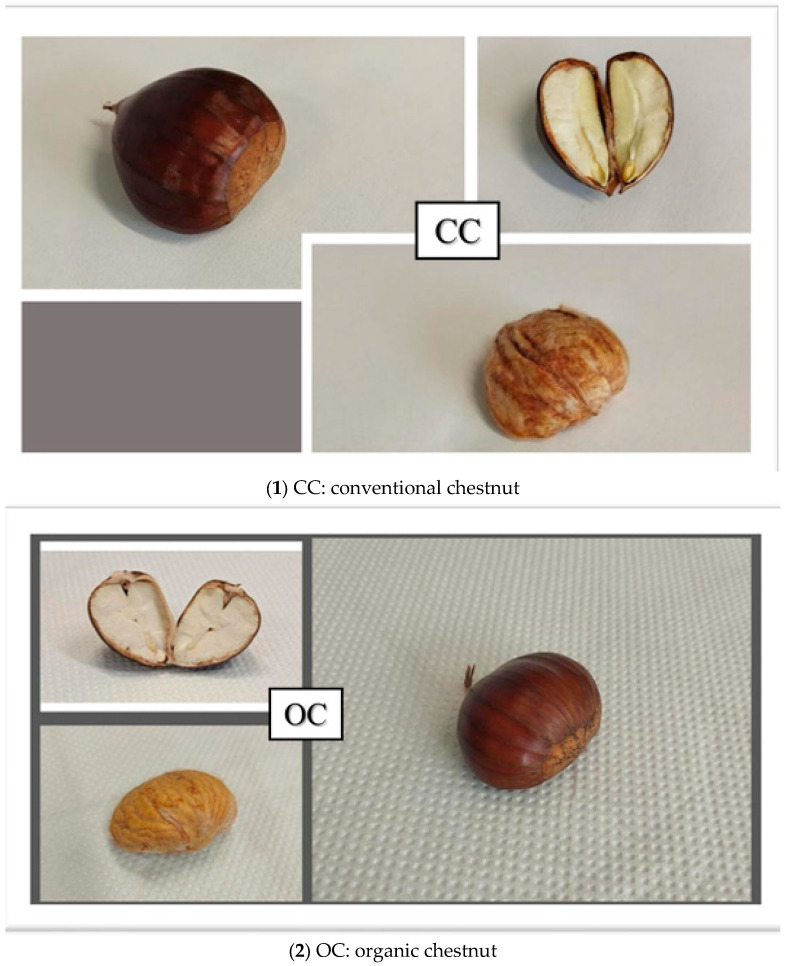
Images of the analyzed chestnut samples.

Chestnut samples of the Marron cultivar, both organic and conventional (5 kg for each type), were collected in October 2024 in the area of the Monti Cimini (42°23′06″ N, 12°15′53″ E), in Vallerano (province of Viterbo, central Italy). The chestnuts were grown by Mastrogregori Aldo S.r.l., located in Monti Cimini. The fruits were manually selected to eliminate any damaged ones. The chestnuts were separated into two batches for each type. The first batch (1 kg) was used for chemical analysis, while the second batch (4 kg) was used for sensory analysis. The samples intended for the sensory analysis were provided to the trained panelists for the tasting sessions (see Section 2.4). Organic chestnuts (OCs) and conventional chestnuts (CCs) were analyzed (Figure 1). The samples came from a single geographical area with the same climate. The same cultural practices were applied to both organic and conventional chestnut orchards, with one key exception: in the organic orchards, micronutrients (nitrogen, calcium, and magnesium) were used for fertilization, and sulfur-based products were applied for pest control.

### 2.2. Chemicals and Reagents

The chemicals and reagents used in this study were of analytical grade and obtained from Merck KGaA (Darmstadt, Germany) unless stated otherwise.

### 2.3. Morphological Features

Fifty chestnuts were randomly sampled to assess the morphological characteristics of length, width, thickness, geometric mean diameter, sphericity, volume, and area [14,15]. Dimensions of each chestnut (length, L; width, W; thickness, T) were measured using digital calipers with 0.01 mm resolution. The geometric mean diameter (Dg), sphericity (Ø), arithmetic mean diameter (Da), area (A), and volume (V) were measured according to Mohsenin [16].

### 2.4. Sensory Evaluation

A screening process was undergone by all participants to ensure that no nut allergies were present, that none were pregnant, and that none were smokers. All participants were given the chance to say yes to taking part, and everyone did so by signing the consent form (see the Supplementary Materials for more on this). The sensory research was carried out in two ways: firstly, a panel of trained tasters was used to identify the sensory profiles of the products; secondly, a consumer survey was conducted using the Check-All-That-Apply method (CATA). Regarding the trained panel, 8 persons (4 men and 4 women, between 27 and 65 years of age), trained and led by a panel leader, performed the sensory evaluation in accordance with ISO 8589 [17] and UNI EN ISO 13299 [18] standards. All procedures in the studies on human subjects were in accordance with the ISO and UNI EN ISO standards for ethical requirements in organoleptic analysis. In addition, the sensory analysis was carried out by a panel officially recognized by the Ministry of Agriculture, Food Sovereignty and Forestry (Italy) and informed consent was obtained from each subject prior to their participation in the study. The tests were carried out between the hours of 16:00 and 18:00 in a laboratory for sensory analysis. The chestnut samples, both organic (OC) and conventional (CC), were prepared anonymously and were presented in white Pyrex dishes. Mineral water was at the disposal of the panel members for palate cleansing. As in our previous research [19], we chose to conduct sensory assessments on raw chestnuts to avoid any possible influence of cooking treatments. Two group sessions were held in which panel members worked to develop a vocabulary of descriptive terms to be used. In the third session, each panelist evaluated the samples individually in their own tasting booth. Table 1 shows the descriptors, definitions, and associated reference standards used [13,20,21]. The score for each descriptor was scored from 0 (absence of the descriptor) to 10 (maximum intensity of the descriptor) [22]. Lastly, the overall judgment of each panel member was added based on the same scale.

The CATA (Check-All-That-Apply) test was conducted using the selected attributes. This method is quick and straightforward, making it effective for gathering consumer opinions [23]. A total of 100 untrained participants (55 women and 45 men, aged 25–65 years) took part in the study. Only healthy volunteers with no history of food allergies, no known issues with taste or odor, and no alcohol intolerance were included.

Participants underwent a brief training session on the experimental procedure and the proper use of the CATA questionnaire [24]. Each participant received five chestnuts, both organic and conventional, in a white plastic bowl and was instructed to observe and smell each sample before tasting them. After tasting, participants completed the CATA questionnaire, which included the following descriptors: ease of peeling, crunchiness, sweetness, bitterness, astringency, chestnut aroma, hazelnut aroma, almond aroma, butter aroma, caramel aroma, and floral aroma.

Finally, participants were asked to indicate their preference for each sample and to rate its acceptability using a 10-point hedonic scale (1 = “extremely disliked”, 10 = “extremely liked”).

### 2.5. Total Phenolic Compound (TPC) Content and Total Antioxidant Capacity (TAC) Determination

#### 2.5.1. Extracts’ Preparation for Polyphenol Compounds and Antioxidant Activity Determination

For the analysis of the total phenolic compounds and antioxidant activity, samples were extracted according to Costantini et al. [25]. Organic chestnuts without pellicle (OCs) and with pellicle (OCPs) were analyzed in comparison with conventional chestnuts without pellicle (CCs) and with pellicle (CCPs). Briefly, all samples were ground in a laboratory mill (IKA^®^ A11 basic Analytical mill (IKA^®^-Werke GmbH & CO., KG, Staufen im Breisgau, Germany), and ground samples were extracted overnight in the dark with 80% aqueous methanol (1:25, *w*/*v*). Then, the samples were centrifuged at 10.000 rpm (ALC PK121R centrifuge; Bodanchimica s.r.l., Cagliari, Italy) for 10 min at 4 °C. The supernatant was collected and used for analysis.

#### 2.5.2. Total Phenolic Compound (TPC) Content

The TPC content was determined using the Folin–Ciocalteu standard method as modified by Costantini et al. (2014) [25] and adapted for 96-well plates and an automatic reader (Infinite 2000, Tecan, Salzburg, Austria). Briefly, 30 µL of deionized water was added to 10 µL of ethanolic extract, 10 µL of Folin–Ciocalteu reagent, and 200 µL of 30% Na_2_CO_3_. The absorbance of the mixture was measured at 725 nm on a plate reader (Infinite F200, TECAN, Männedorf, Switzerland). A gallic acid standard curve was prepared and the results were expressed as mg of gallic acid equivalents (GAE)/g of the sample.

#### 2.5.3. Total Antioxidant Capacity (TAC) Determination

The TAC was assessed by ferric reducing antioxidant power (FRAP) and 2,2′-azino-bis (3-ethyl-benzothiazoline-6-sulfonic acid) (ABTS^•+^) radical scavenging activity assays as described as follows.

FRAP assay was performed using the method described by Benzie and Strain [26], which was adapted for 96-well plates and an automatic reader (Infinite 2000, Tecan, Salzburg, Austria). The method is based on the reduction of the Fe^3+^-2,4,6-tripyridyl-s-triazine (TPTZ) complex to its ferrous form at a low pH. Briefly, 160 µL of FRAP assay solution (consisting of 20 mM ferric chloride solution, 10 mM TPTZ solution, and 0.3 M acetate buffer at pH 3.6) was prepared daily, mixed with 10 µL of the sample, standard, or blank, and dispensed into each well of a 96-well plate. The absorbance was measured at 595 nm at 37 °C after 30 min of incubation. The results were expressed as mmol Fe^2+^ equivalents/g.

The ABTS^•+^ radical scavenging activity was evaluated by the OxiSelect^TM^ Trolox Equivalent Antioxidant Capacity (TEAC) Assay Kit (ABTS) (Cell Biolabs INC., San Diego, CA, USA) following the manufacturer’s instructions. The absorbance was recorded at 405 nm in an automatic reader (Infinite 2000, Tecan, Salzburg, Austria). A standard curve for Trolox was prepared and the antioxidant capacity was expressed as mmol of Trolox equivalents (TE)/g.

### 2.6. HS-SPME/GC-MS Analysis of Volatile Chemical Composition

Following Mujic et al. [27], the method for determining the volatile profile was modified slightly. The volatile chemical fraction of chestnuts with and without pellicle samples was characterized by using the Headspace Solid Phase Microextraction (HS-SPME) sampling technique followed by GC/MS analysis. For the extraction, approximately 1 mg of each ground sample was placed inside a 7 mL glass vial with a PTFE-coated silicone septum and a NaCl solution (25% (*w*/*v*) was added. An SPME device from Supelco (Bellefonte, PA, USA) with 1 cm fiber coated with 50/30 μm DVB/CAR/PDMS (divinylbenzene/carboxen/polydimethylsiloxane) was used to capture the volatile components. After reaching the equilibration phase, the fiber was exposed to the headspace of the sample for 30 min at 40 °C. Then, to desorb the volatiles, the SPME fiber was inserted in the GC injector maintained at 250 °C for two minutes, in splitless mode. The analyses were carried out by a gas chromatograph coupled with a mass spectrometer Clarus 500 model Perkin Elmer (Waltham, MA, USA), equipped with an FID (flame ionization detector). A Varian Factor Four VF-5 was used to separate the analytes. The temperature program was as follows: 40 °C held for 2.0 min and then increased by 6 °C per minute up to 220° for 10 min. Helium was used as a gas carrier at a constant rate of 1.0 mL min^−1^.

The mass spectrometer was operated at 70 eV (EI) in full scan mode in the range 35–450 *m*/*z*. Ion source and the connection parts temperature were set a 180 °C and 200 °C, respectively. The identification of the volatiles was performed by matching their mass spectra with those stored in the Wiley 2.2 and NIST 11 mass spectra libraries (Scientific Instrument Services (SIS). In addition, the Linear Retention Indices (LRIs) were calculated using a series of n-alkanes standard (C_8_–C_30_) analyzed under the same conditions and compared with those available in the literature. For the quantification procedure, the peak areas of the FID signal were used to calculate the relative concentrations expressed as percentages without the use of an internal standard or any factor correction. All analyses were carried out in triplicate.

### 2.7. Statistical Analysis

Statistical analysis was conducted using XLSTAT 2024.1.1 software (Addinsoft SARL, New York, NY, USA) with a one-way analysis of variance (ANOVA). Fisher’s least significant difference (LSD) test was applied to determine statistical differences between means at a significance level of *p* < 0.05.

## 3. Results

### 3.1. Morphological Features

The morphological characteristics studied are shown in Table 2. It can be observed that the parameters of organic chestnuts are significantly different from those of the conventional chestnuts. OCs, organic chestnuts, have a slightly higher fruit weight (19.57 ± 0.18 g versus 19.25 ± 0.20 g for CCs), as well as greater length, width, and thickness. However, both organic and conventional samples can be classified as large nuts, as both require 50 nuts to produce 1 kg of nuts [28,29]. Regarding the sphericity parameter, there were no significant differences between the samples.

### 3.2. Sensory Evaluation

Table 3 presents the sensory attributes of organic and conventional chestnuts. The trained panel described organic chestnuts as having several distinctive sensory characteristics that set them apart from conventional chestnuts. Specifically, organic chestnuts exhibit a distinct aroma pattern compared to their conventional counterparts. Among the sensory attributes of organic chestnuts (OCs) compared to conventional chestnuts (CCs) (9 vs. 8), hazelnut (5.87 vs. 5), almond (5 vs. 4), butter (3.96 vs. 3), and floral (5.95 vs. 4.96) aromas are significantly higher. Organic chestnuts are strongly characterized by a caramel aroma. Conventional chestnuts do not have this flavor at all (1.95 vs. 0). The significant presence of a caramel aroma was already reported in our recent study [19]. In addition, organic chestnuts had a significantly higher crunchiness score than conventional chestnuts (9.02 vs. 7.96). This result can be seen as an advantage for organic samples in terms of quality. In fact, some researchers have shown how the sound of crunchiness in the mouth is perceived by the consumer as a synonym of quality [30]. More importantly, organic chestnuts had a higher aromatic intensity than conventional chestnuts (8.81 vs. 7.97) and received a significantly higher overall judgment from panel members (8.97 vs. 8.47).

It is noteworthy that the organic chestnuts received a higher score in the overall evaluation by the panel (8.97), which is close to the score observed by Frangipane et al. [13] for the cv Marrone (9.08), considered to be of excellent quality. Moreover, in the case of organic chestnuts (OCs), the degree of penetration of the pellicle into the kernel is zero (Table 3). This result is another argument for the quality of the organic samples. In fact, not only in the fresh market, but also in the confectionery market, it is a characteristic that increases the value of the use of chestnuts [31]. The correlation analysis presented in Table 4 allows us to observe that in the case of organic chestnuts, the overall judgment shows a positive correlation with crunchiness (Pearson’s coefficient = 0.77). This confirms that the crunchiness attribute strongly influences the perception of chestnut quality. Likewise, a caramel aroma and aromatic intensity also have a moderate positive correlation with the overall judgment (Pearson’s coefficient = 0.429 and 0.238, respectively), indicating that both contribute positively to the preferences of the panelists.

**Figure 2:**
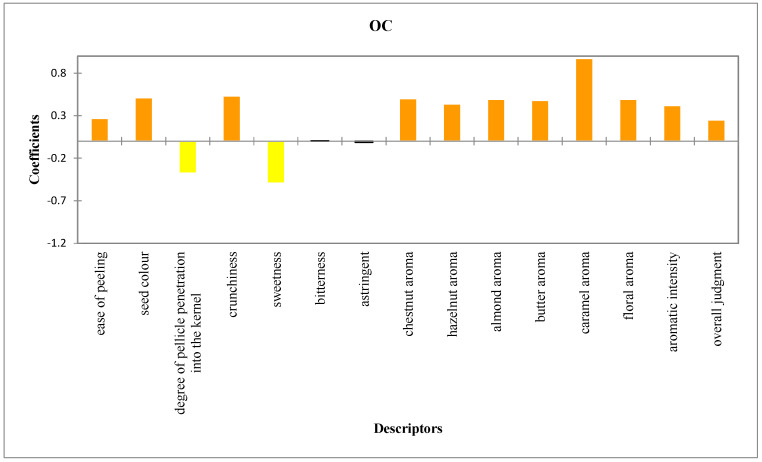
Coefficients of the models of analysis of variance for characterizing the product according to the descriptors in organic chestnuts (OCs).

**Figure 3:**
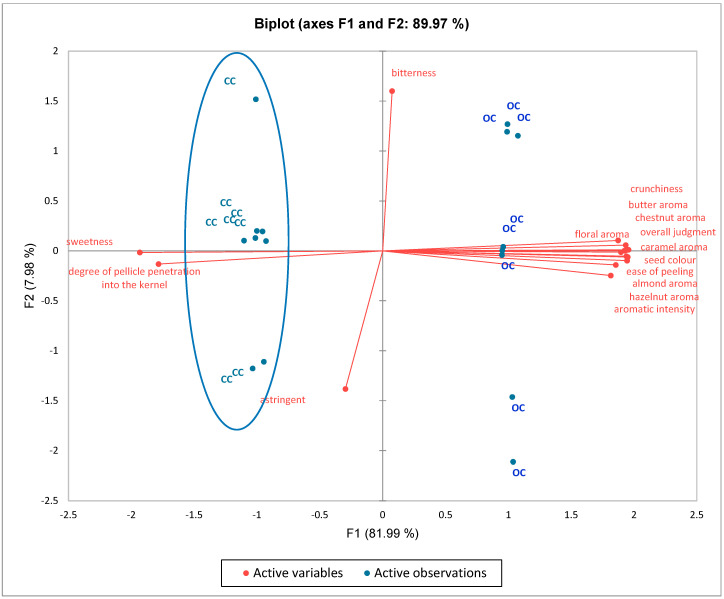
PCA loading plot with multivariate variation for sensory attributes and overall judgment between organic (OC) and conventional (CC) chestnut samples.

**Figure 4:**
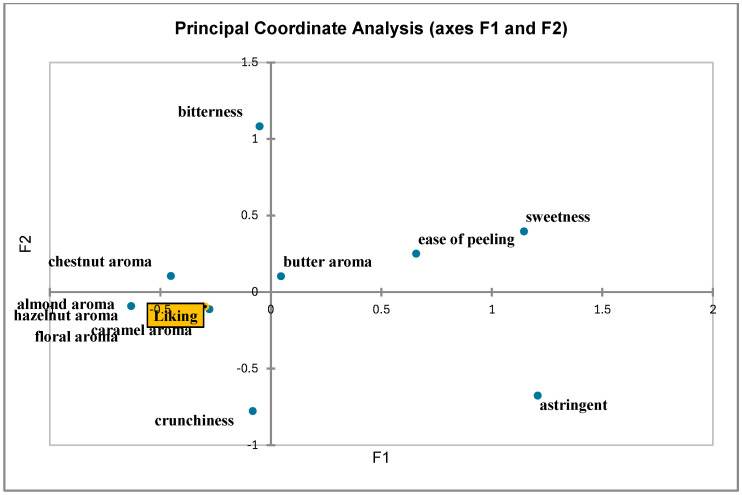
The CATA (Check-All-That-Apply) test with the opinions of consumers on the analyzed samples of chestnuts.

Through statistical sensory data analysis, we identified the descriptors that best distinguish the product. An orange color is linked to coefficients with a significantly positive value, while a yellow color is associated with coefficients with a significantly negative value. Notably, as shown in Figure 2, the caramel aroma is the descriptor most strongly associated with organic chestnuts. Conversely, sweetness and the degree of pellicle penetration into the kernel did not characterize the organic samples. A Principal Component Analysis (PCA) was also conducted to describe the sensory differences and the overall evaluation by the panel. As shown in Figure 3, the first two components account for 89.97% of the total variance (F1 = 81.99%; F2 = 7.98%), clearly distinguishing organic and conventional chestnuts at the sensory level. The attributes contributing most to F1 were crunchiness, overall rating, aromatic intensity, and the aromas of chestnut, butter, floral, caramel, almond, and hazelnut. In contrast, bitterness was the attribute most strongly correlated with F2. As illustrated in the figure, the organic (OC) samples are positioned in the upper and lower right quadrants, characterized by pronounced aromas of chestnut, hazelnut, almond, butter, caramel, and floral notes. Conventional chestnuts (CCs), on the other hand, are situated in the lower and upper left quadrants. They are characterized by astringency, sweetness, and the degree of penetration of the skin into the kernel. It must be noted that this last characteristic contributes to their poor quality. The results of the CATA test, which was conducted with 100 participants, are summarized in Figure 4. On the basis of the data obtained from the CATA test [32], the influence of the perception of the sensory attributes on the consumers’ liking of organic and conventional chestnuts was studied. For this purpose, a principal coordinate analysis was performed on the correlation coefficients and the results were visualized on 2D maps (Figure 4). In particular, the results show that consumer preference was closely associated with caramel, floral, hazelnut, almond, and chestnut attributes. It is important to note that these attributes are specific and characteristic of organic chestnuts. Therefore, it can be concluded that the consumers preferred the attributes related to organic chestnuts, showing their liking for these samples. As a result, the samples of organic chestnuts were preferable to the conventional ones, both by the consumers and by the trained panel.

### 3.3. Total Phenolic Compounds (TPC) and Total Antioxidant Capacity (TAC)

**Figure 5:**
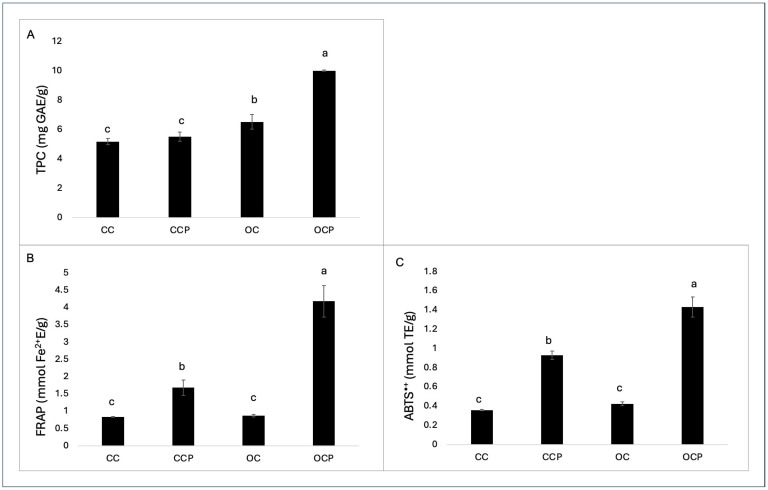
(**A**) Total phenolic content (TPC) (mg GAE/g); (**B**) ferric reducing antioxidant power assay (FRAP) (mmol Fe^2+^E/g); (**C**) ABTS^•+^ radical scavenging activity (mmol TE/g). Data represents mean ± standard deviation of n = 3 biological replicates and n = 2 technical replicates. Different letters indicate significant differences (Fisher’s test, *p* ≤ 0.05) in each analysis, according to analysis of variance. CC: conventional chestnut; CCP: conventional chestnut with pellicle; OC: organic chestnut; and OCP: organic chestnut with pellicle.

Figure 5 shows the values of total phenolic compounds (TPCs) and antioxidant activity, determined by the FRAP and ABTS^•+^ tests. These values were obtained for organic and conventional chestnut samples, both with and without pellicle. TPC and antioxidant capacity differed significantly. Compared to CC samples (5.18 mg GAE/g), TPC values were highest in OC samples (6.54 mg GAE/g). It is noteworthy that the TPC values in the samples with pellicle were about twice as high in organic chestnuts (OCPs) as in conventional chestnuts (CCPs) (10.02 vs. 5.18 mg GAE/g, respectively). One observation in this regard is that organic farming does not use chemical pesticides. This could induce defense mechanisms against pathogen attack with a consequent increase in the biosynthesis of phenolic compounds [33]. Overall, the TPC values in the chestnut samples we analyzed are higher than those previously found in several cultivars [34,35,36]. Moreover, organic chestnuts with pellicle (OCPs) showed significantly higher values in terms of antioxidant capacity with 1.43 mmol TE/g (ABTS^•+^) and 4.18 mmol Fe^2+^E/g (FRAP) compared to conventional chestnuts with pellicle (CCPs) with 0.93 mmol TE/g (ABTS^•+^) and 4.18 mmol Fe^2+^E/g (FRAP). This is particularly important as it opens new perspectives for using chestnut pellicle as a potential antioxidant resource and natural additive in functional foods [37]. The organic and conventional samples exhibited different antioxidant activity values, measured using FRAP and ABTS methods, displaying a pattern analogous to that seen in total polyphenol content (TPC) levels. Our research revealed an excellent correlation coefficient between total phenolic content and antioxidant activity indicating that phenolic compounds are the primary antioxidants in chestnuts. Nevertheless, establishing the specific role of individual bioactive compounds in total antioxidant activity is complicated because of the interaction between the various substances [8,11]. Overall, our study showed a higher antioxidant capacity in the chestnuts analyzed in comparison with previous research [34]. Earlier studies have reported higher total phenols and antioxidant capacity in organic fruits and vegetables compared to conventional ones [38]. Nevertheless, we are not aware of any studies investigating the polyphenol content and antioxidant capacity of organic and conventional chestnuts.

### 3.4. Volatile Chemical Composition

Concentrations of volatile compounds calculated from SPME headspace analysis peak areas (%) for organic and conventional chestnuts are reported in Table 5. Qualitative differences were found between chestnuts from organic and conventional farming. In particular, in organic chestnuts (OCs), the volatile profile was characterized by 2-propanol (2.2%), reminiscent of butter notes [27]. This was confirmed by the results of the sensory analysis (Table 3), in which the butter aroma was significantly predominant in organic samples compared to conventional ones. The main volatile compounds characterizing organic chestnuts were also 2-pentanol (1.1%), 2-pentanone (1.5%, with a fruity flavor), 3-methyl-1-butanol (3.3%), 2-methyl-1-butanol (5.1%), and 3-octanol (5.5%). An interesting result was obtained for organic chestnuts with and without pellicle (OCPs and OCs, respectively). In fact, decanal and 1-pentanol were exclusively present in these samples and were absent in conventional chestnuts with and without pellicle (CCPs and CPs, respectively). Indeed, as previously observed [27], 1-pentanol imparts vanilla and caramel notes when it is present. This could be precisely related to the exclusive presence of caramel aromatic notes detected by sensory analysis (Table 3) in organic samples compared to conventional ones (1.95 vs. 0.00). Quantitatively, octanal was more abundant in conventional chestnuts without pellicle (CCs) than organic chestnuts without pellicle (OCs) (10.6% vs. 0.2%, respectively) as well as nonanal (7.5% vs. 5.5%, respectively). Moreover, from a quantitative point of view, 1-octanol emerged as the most abundant component in both organic and conventional chestnuts. More precisely, it predominates in conventional chestnuts with pellicle (CCP with 27.8%) compared to organic chestnuts with pellicle (OCP with 25%). Finally, in agreement with recent research [39], the presence of 1-hexanol, characterized by a pleasant floral odor, was found in all samples, both with and without pellicle. Organic chestnuts without pellicle (OC) showed a higher percentage of 1-hexanol than conventional chestnuts without pellicle (CC) (7.1 vs. 5.5%). On the other hand, also in sensory analysis, panelists found more floral aroma in organic samples (Table 3).

## 4. Conclusions

In conclusion, this research highlights the profound difference in the sensory profile, volatile composition, and antioxidant capacity of chestnuts from organic farming compared to those from conventional farming. The key findings of the study were that organic chestnuts have higher phenolic content and antioxidant activity than conventional chestnuts. In addition, organic chestnuts have a different sensory profile compared to the conventional chestnuts. Our study found that the relationships between attributes and consumers’ perceived liking revealed a strong association between liking and the attributes of caramel, floral, hazelnut, almond, and chestnut. It is important to note that these attributes are specific to and characteristic of organic chestnuts. As a result, both consumers and the trained panel prefer organic chestnut samples over the conventional ones. We acknowledge a limitation of this study: the lack of sample analysis across multiple production years; therefore, future research should include multi-year sampling to provide a more comprehensive assessment. A comparison of conventional and organic practices reveals the necessity of making trade-offs between the various evaluated effects. Conventional cultivation has a negative impact due to the toxicity of the pesticides used; however, it has a higher yield than organic production. On the other hand, the preliminary findings presented here provide valuable insights into the sensory characteristics of organic chestnuts and reinforce their role under environmental sustainability and consumer health protection. Ultimately, promoting the consumption of organic chestnuts by enhancing knowledge and awareness of their characteristics can foster their use, contributing to important health and environmental sustainability goals.

## Figures and Tables

**Table 1:** Descriptions of sensory attributes and associated reference standards for chestnuts [12,15].

Descriptors	Sensory Attribute Definitions	Standards and Reference Materials
ease of peeling	the ease of peeling the shell and pellicle away from the nut	different levels of adherence of shell/pellicle to the nut (a value of 0 corresponds to hard, while 10 corresponds to easy)
seed color	the external color of the seed, after removing the pellicle	seed color with a degree of darkness (value 0 corresponds to a light colour seed, while value 10 corresponds to dark)
degree of pellicle penetration into the kernel	the degree of penetration of the seed coat into the embryo	a value of 0 corresponds to no penetration, while 10 corresponds to strong penetration (visible > 2.0 mm)
crunchiness	the amount of noise generated when the sample is chewed at a fast rate with the back teeth	0 corresponds to a dried apple piece, while 10 corresponds to a fresh celery piece
astringency	the sensation of drying, drawing-up, or puckering of any of the mouth surfaces	diluted tannic acid solution (0.06–2 mg/mL)
sweetness	the basic taste associated with sugar (sucrose)	diluted sucrose solution (0.5–6 g/L)
bitterness	the basic taste associated with caffeine	diluted caffeine solution (0.03–0.2 g/L)
chestnut aroma	intensity of aroma of chestnut products	taste of chestnut
hazelnut aroma	sweet, oily, somewhat woody aromatics associated with hazelnuts	taste of hazelnut
almond aroma	sweet cherry pit-like nutty aromatics associated with almonds	taste of almond
butter aroma	aromatics commonly associated with natural, slightly salted butter	taste of butter
caramel aroma	aromatics associated with caramel	taste of caramel
floral aroma	aromatics associated with flowers	taste of floral
walnut aroma	aromatics associated with walnuts	taste of walnut
citrus aroma	aromatics associated with citrus fruits	taste of citrus
wood/musk	aromatics associated with wood or musk	taste of woody
aromatic intensity	the characteristic flavor of the chestnut at the seed break	aromatics commonly associated with chestnut

**Table 2:** The analysis of the morphological characteristics of the analyzed organic and conventional chestnuts.

Samples	Weight (g)	Length (mm)	Width (mm)	Thickness (mm)	Geometric Mean Diameter (mm)	Arithmetic Mean Diameter (mm)	Surface Area (mm^2^)	Sphericity (%)	Volume (m^3^)
OC	19.57 ± 0.18 ^a^	40.37 ± 0.22 ^a^	32.32 ± 0.20 ^a^	23.32 ± 0.19 ^a^	31.11 ± 0.18 ^a^	32.00 ± 0.18 ^a^	3040.34 ± 36.37 ^a^	0.77 ± 0.01 ^a^	15,921.26 ± 284.60 ^a^
CC	19.25 ± 0.20 ^b^	40.02 ± 0.24 ^b^	32.05 ± 0.21 ^b^	23.00 ± 0.18 ^b^	30.79 ± 0.18 ^b^	31.69 ± 0.19 ^b^	2977.64 ± 36.32 ^b^	0.76 ± 0.01 ^a^	15,430.80 ± 284.58 ^b^
Pr > F(Model)	0.001	0.012	0.042	0.002	0.001	0.001	0.001	0.274	0.001
Significant	Yes	Yes	Yes	Yes	Yes	Yes	Yes	No	Yes

CC: conventional chestnut; OC: organic chestnut. Note: Means that show different letters are significantly different (*p* < 0.05).

**Table 3:** Least Squares Means of sensory attributes and overall judgment of the organic and conventional chestnuts analyzed by the trained panel.

Sensory Attributes	Organic Chesnut (OC)	Conventional Chestnut (CC)
ease of peeling	5.54 ± 0.28 ^a^	5.00 ± 0.20 ^b^
seed color	6.00 ± 0.53 ^a^	4.98 ± 0.50 ^b^
degree of pellicle penetration into the kernel	0.00 ± 0.00 ^a^	0.75 ± 0.42 ^b^
crunchiness	9.03 ± 0.57 ^a^	7.96 ± 0.51 ^b^
sweetness	6.98 ± 0.48 ^a^	7.96 ± 0.50 ^b^
bitterness	0.94 ± 0.11 ^a^	0.93 ± 0.10 ^b^
astringency	0.93 ± 0.09 ^a^	0.95 ± 0.10 ^b^
chestnut aroma	9.00 ± 0.52 ^a^	8.00 ± 0.50 ^b^
hazelnut aroma	5.88 ± 0.47 ^a^	5.00 ± 0.45 ^b^
almond aroma	5.00 ± 0.51 ^a^	4.01 ± 0.50 ^b^
butter aroma	3.96 ± 0.50 ^a^	3.00 ± 0.46 ^b^
caramel aroma	1.95 ± 0.98 ^a^	0.00 ± 0.00 ^b^
floral aroma	5.95 ± 0.51 ^a^	4.96 ± 0.41 ^b^
aromatic intensity	8.81 ± 0.47 ^a^	7.98 ± 0.40 ^b^
overall judgment	8.98 ± 0.26 ^a^	8.48 ± 0.23 ^b^

Note: Means that show different letters are significantly different (*p* < 0.05).

**Table 4:** Pearson’s coefficient in organic chestnut (OC) samples.

Attributes	Floral Aroma	Astringency	Caramel Aroma	Sweetness	Hazelnut Aroma	Almond Aroma	Bitterness	Ease of Peeling	Butter Aroma	Aromatic Intensity	Crunchiness	Overall Judgment
floral aroma	**1**	0.200	0.111	0.048	0.111	0.333	0.207	0.022	0.005	0.022	0.005	0.048
astringency	0.200	**1**	0.200	0.086	0.022	0.000	0.190	0.004	0.052	0.004	0.009	0.086
caramel aroma	0.111	0.200	**1**	0.429	0.111	0.333	0.105	0.200	0.048	0.022	0.403	0.429
sweetness	0.048	0.086	0.429	**1**	0.429	0.571	0.045	0.086	0.020	0.086	0.002	0.020
hazelnut aroma	0.111	0.022	0.111	0.429	**1**	0.333	0.105	0.022	0.132	0.200	0.005	0.048
almond aroma	0.333	0.000	0.333	0.571	0.333	**1**	0.456	0.267	0.254	0.000	0.000	0.000
bitterness	0.207	0.190	0.105	0.045	0.105	0.456	**1**	0.527	0.219	0.190	0.159	0.045
ease of peeling	0.022	0.004	0.200	0.086	0.022	0.267	0.527	**1**	0.560	0.360	0.224	0.086
butter aroma	0.005	0.052	0.048	0.020	0.132	0.254	0.219	0.560	**1**	0.306	0.002	0.020
aromatic intensity	0.022	0.004	0.022	0.086	0.200	0.000	0.190	0.360	0.306	**1**	0.288	0.238
crunchiness	0.005	0.009	0.403	0.002	0.005	0.000	0.159	0.224	0.002	0.288	**1**	0.770
overall judgment	0.048	0.086	0.429	0.020	0.048	0.000	0.045	0.086	0.020	0.238	0.770	**1**

**Table 5:** Volatile content (percentage mean value ± standard deviation) of biological and conventional chestnuts, as determined by HS-SPME/GC–MS.

N°	Component ^1^	LRI ^2^	LRI ^3^	OCP (%Area)	OC (%Area)	CCP (%Area)	CC (%Area)
1	ethanol	430	436	30.5 ± 1.2 ^a^	22.2 ± 1.3 ^b^	25.5 ± 1.4 ^c,b^	18.2 ± 0.9 ^d^
2	2-propanol	475	481	tr	2.2 ± 0.06 ^a^	-	0.5 ± 0.02 ^b^
3	acetone	505	500	3.5 ± 0.08 ^a^	0.8 ± 0.02 ^b^	2.7 ± 0.05 ^c^	2.0 ± 0.05 ^d,c^
4	2-pentanol	660	664	0.1 ± 0.01 ^a^	1.1 ± 0.06 ^b^	tr	0.5 ± 0.02 ^c^
5	2-pentanone	690	684	1.0 ± 0.03 ^a^	1.5 ± 0.05 ^b,c^	tr	0.2 ± 0.02 ^d^
6	3-methyl-1-butanol	722	719	1.2 ± 0.05 ^a^	3.3 ± 0.06 ^b^	0.5 ± 0.03 ^c^	0.9 ± 0.04 ^d^
7	2-methyl-1-butanol	746	744	1.8 ± 0.04 ^a^	5.1 ± 0.05 ^b^	1.1 ± 0.04 ^c^	1.9 ± 0.04 ^d,a^
8	1-hexanol	800	801	10.5 ± 0.09 ^a^	7.1 ± 0.10 ^b^	9.9 ± 0.14 ^c,a^	5.5 ± 1.60 ^d^
9	2-heptanone	865	873	8.1 ± 0.20 ^a^	3.2 ± 0.04 ^b^	9.6 ± 0.15 ^c^	5.1 ± 0.05 ^d^
10	benzaldehyde	918	923	0.8 ± 0.04 ^a^	0.2 ± 0.02 ^b^	1.1 ± 0.04 ^c^	0.5 ± 0.02 ^d,b^
11	1-octen-3-ol	975	980	tr	0.5 ± 0.02 ^a^	-	0.1 ± 0.01 ^b^
12	3-octanol	993	997	1.2 ± 0.05 ^a^	5.5 ± 0.06 ^b^	1.5 ± 0.04 ^c,a^	4.0 ± 0.06 ^d^
13	limonene	1018	1026	2.3 ± 0.05 ^a^	1.1 ± 0.03 ^b^	1.8 ± 0.05 ^c^	0.6 ± 0.03 ^d^
14	1-octanol	1075	1080	25.0 ± 2.0 ^a^	20.2 ± 2.5 ^b^	27.8 ± 1.8 ^c^	22.1 ± 2.1 ^d^
15	2-nonanone	1089	1091	0.8 ± 0.03 ^a^	tr	0.1 ± 0.01 ^b^	-
16	(*E*)-2-hexenal	1200	1205	1.2 ± 0.07 ^a^	1.1 ± 0.05 ^a^	0.9 ± 0.03 b	0.2 ± 0.12 ^c^
17	decanal	1207	1217	0.9 ± 0.05 a	0.2 ± 0.02 ^b^	-	-
18	1-pentanol	1230	1233	0.1 ± 0.01 ^a^	0.5 ± 0.02 ^b^	-	-
19	octanal	1285	1278	0.5 ± 0.03 ^a^	0.2 ± 0.02 ^b^	3.9 ± 0.04 ^c^	10.6 ± 0.09 ^d^
20	2-heptanol	1333	1325	tr	1.8 ± 0.05 ^a^	1.7 ± 0.07 ^a^	3.9 ± 0.09 ^b^
21	nonanal	1387	1390	0.8 ± 0.03 ^a^	5.5 ± 0.07 ^b^	2.7 ± 0.05 ^c^	7.5 ± 0.06 ^d^
22	acetic acid	1420	1427	9.2 ± 0.08 ^a^	15.2 ± 0.2 ^b^	7.8 ± 0.05 ^c^	15.3 ± 0.1 ^d,b^
	SUM			99.5	98.5	98.6	99.6

Legend: ^1^ The components are reported according to their elution order on VF-5 column. ^2^ Linear Retention Indices measured on VF-5 column. ^3^ Linear Retention Indices from the literature. Data are means ± standard deviation of three (n = 3) replicates. Means with different letters among the columns are significantly different (ANOVA test followed by Tukey’s HSD test, *p* < 0.01). OCP: organic chestnut with pellicle; OC: organic chestnut; CCP: conventional chestnut with pellicle; CC: conventional chestnut; Tr: percentage mean values < 0.1%; and -: not detected.

## Data Availability

The data presented in this study are available on request from the co-author, Maria Teresa Frangipane.

## Supplementary Materials

The following supporting information can be downloaded at: https://www.mdpi.com/article/10.3390/foods14122013/s1, File S1: Informed Consent Form for Sensory Evaluation of organic and conventional chestnuts.
