# Supplementary material for: Organic vs. Conventional Chestnuts (Castanea sativa Mill.): A Focus on Antioxidant Activity, Volatile Compounds, and Sensory Profile
Source: Foods. 2025 Jun 6;14(12):2013. doi: 10.3390/foods14122013 (PMC12191601; doi:10.3390/foods14122013)
Supplement: Supplementary file 1 [file foods-14-02013-s001.zip › foods-3666604-Supplementary Materials.pdf]

Supplementary Materials

Informed Consent Form for Sensory Evaluation of organic and conventional chestnuts

Purpose of the Study

In this sensory evaluation, chestnuts that have been organically grown are tasted alongside those that have been conventionally grown. Participation is voluntary; however, we would like to inform you that by participating you will contribute to the complete outcome of the research.

Eligibility Criteria

For safety reasons, individuals who meet any of the following criteria are not eligible to participate in this sensory evaluation:

- Individuals with food allergies
- Pregnant or lactating women
- Minors (under the age of 18)

Data Protection

Rest assured that all the personal information provided will be kept strictly confidential. Without explicit permission being granted by the researchers, details regarding this evaluation are not to be disclosed by you.

Participant Declaration

I confirm that I am in good health and not affected by any of the above conditions. I am therefore fit to participate in this sensory evaluation of chestnuts.

Voluntary Participation

By providing my personal data and signature below, I voluntarily indicate my informed consent to participate in this study. Furthermore, I may withdraw from studying at any time without penalty.

Participant Information

Name: ________________________
Signature: _____________________
Date: _________________________
